# Use of electronic patient data overview with alerts in primary care increases prescribing of lipid-lowering medications in patients with type 2 diabetes

**DOI:** 10.1007/s00125-021-05598-x

**Published:** 2021-10-28

**Authors:** Morten H. Charles, Janus L. Thomsen, Bo Christensen, Ryan Pulleyblank, Line P. Kongstad, Kim Rose Olsen

**Affiliations:** 1 Department of Public Health, Research Unit for General Practice, Aarhus University, Aarhus, Denmark; 2 Steno Diabetes Center, Aarhus, Denmark; 3 Center for General Practice at Aalborg University, Aalborg, Denmark; 4 Research Unit for General Practice, Aarhus University, Aarhus, Denmark; 5 Danish Centre for Health Economics, Department of Public Health, University of Southern Denmark, Odense, Denmark

**Keywords:** Alerts, Disease management, Electronic health records, General practice

## Abstract

**Aims/hypothesis:**

We aimed to assess whether general practices (GPs) using an electronic disease management program (DMP) with population overviews, including alerts when patients failed to receive guideline-recommended prescription medications, increased prescriptions of lipid-lowering drugs for patients with type 2 diabetes with no history of lipid-lowering treatment.

**Methods:**

This observational study included 165 GPs that reached a high level of use of the DMP in 2012 and a control group of 135 GPs who reached a high level of use in 2013 and, hence, who were less exposed to the DMP throughout 2012. A binary measure for having been prescribed and filled lipid-lowering drugs at any time within a 12-month exposure period was derived for all patients with type 2 diabetes who did not receive a prescription for lipid-lowering drugs in the baseline year prior to the study period (i.e. 2011). Results were derived using ORs from multivariate logistic regression analyses. Subgroup stratification based on age, sex, diabetes duration, deprivation status and Charlson Comorbidity Index (CCI) score was conducted and assessed. Placebo tests were carried out to assess bias from selection to treatment.

**Results:**

Patients who did not receive a prescription of lipid-lowering drugs in the year prior to being listed with GPs that used the DMP had statistically significant greater odds of receiving a prescription of lipid-lowering medications when compared with individuals who attended control GPs (OR 1.23 [95% CI 1.09, 1.38]). When the analysis period was shifted back by 2 years, no significant differences in lipid-lowering drug prescription between the two groups were found to occur, which indicates that these results were not driven by selection bias. Subgroup analyses showed that the increase in lipid-lowering drug prescriptions was primarily driven by changes among male participants (OR 1.32 [95% CI 1.12, 1.54]), patients aged 60–70 years (OR 1.40 [95% CI 1.13, 1.74]), patients with a diabetes duration of ≤5 years (OR 1.33 [95% CI 1.13, 1.56]), non-deprived patients (OR 1.25 [95% CI 1.08, 1.45]) and patients without comorbidities (CCI score = 0; OR 1.27 [95% CI 1.11, 1.45]).

**Conclusions/interpretation:**

Access to population overviews using a DMP with alerts of clinical performance measures with regard to adhering to guideline-recommended prescription of medications can increase GP prescriptions of lipid-lowering drugs.

**Graphical abstract:**

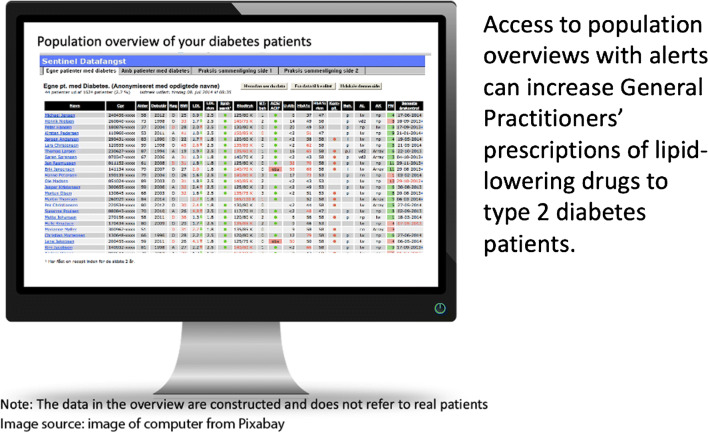

**Supplementary Information:**

The online version contains peer-reviewed but unedited supplementary material available at 10.1007/s00125-021-05598-x.

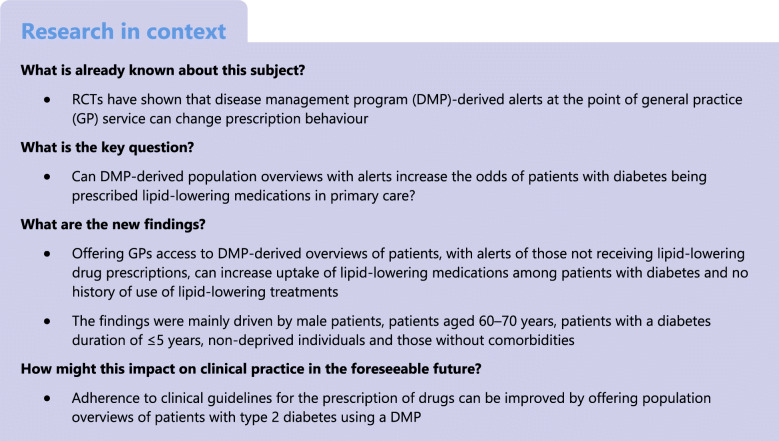



## Introduction

Intensive multifactorial treatment of type 2 diabetes has led to a large decrease in mortality risk among patients with type 2 diabetes. However, many patients are still not administered the recommended treatment and, consequently, do not benefit from possible reductions in the risks of diabetic complications.

The cardioprotective benefits of lipid-lowering treatments are well established among people with clinically diagnosed diabetes. The Danish guidelines are in accordance with the European Association for the Study of Diabetes’ recommendation for pharmacological therapy with lipid-lowering medication for patients with type 2 diabetes at moderate CVD risk. Guidelines recommend an LDL-cholesterol target of <2.6 mmol/l for the majority of patients with type 2 diabetes [1]; however, a large variation in the use of statins in the general population in primary care has been shown [2]. Previous research suggests that the prescribing behaviours of doctors are most likely to contribute to the variation observed [3–5].

In the period from 2011 to 2014, it was possible for Danish general practices (GPs) to use a disease management program (DMP) made available from the Danish Quality Unit of General Practice [6]. The DMP introduced systematic use of electronic health records (EHRs) and presented an overview of all listed patients with diabetes to the GP, including clinical measures, medication and diabetes consultations, to help GPs optimise treatment for their patients. The overview included red flags alerting GPs of patients not receiving treatment as recommended by guidelines [6].

All Danish GPs were obligated to enrol in the system within the initial 3-year period; however, the extent to which GPs used the system in daily clinic/quality assessment was not regulated or financially incentivised, and important differences existed between GPs that practically never used the system and those that used it at a high level. Among other things, these included differences in the historical rates of prescriptions of lipid-lowering medications for patients with type 2 diabetes [6].

The aim of this study was to assess if GPs’ participation in EHR/DMP use increased the probability of GP prescriptions of lipid-lowering drugs for patients with type 2 diabetes with no recent history of lipid-lowering treatment.

## Methods

### Study design

This observational study evaluated the association between GP DMP use and uptake of lipid-lowering medications in a cohort of patients with type 2 diabetes, followed from 2011 to 2013. Approval for conducting the study was provided by the Danish Data Protection Agency (ref. 17/6021). Analyses were conducted between November 2020 and March 2021.

### Study population

The study population was derived from nationwide register data and consisted of adult patients (*N* = 9071) with type 2 diabetes who were not receiving lipid-lowering drugs in the year prior to the study period (i.e. 2011) and who were attending a cohort of 165 ‘exposed’ and 135 ‘control’ GPs (described in detail below). Incident patients were excluded in their index year as they could not have had lipid-lowering prescriptions as part of their diabetes treatment in the year before the study period. Patients were identified algorithmically, based on the Danish Register for Udvalgte Kroniske Sygdomme (RUKS) algorithm [7]. Patients being monitored at the hospital were excluded.

### Data

Data were merged at patient level from the National Patient Register, the National Health Service Register, the Prescription Drug Register and registers with individual socioeconomic variables from Statistics Denmark. Data on GP use of the DMP were obtained from the Danish Quality Unit of General Practice.

### Outcomes

The outcome was measured as a binary variable, with the value being 1 if a patient had received a minimum of one prescription of lipid-lowering medication (anatomical therapeutic chemical [ATC] group C10) within 12 months following the baseline year.

### Treatment exposure

Exposed patient-years consisted of the years that patients attended the 165 ‘exposed’ GPs (GPs which had achieved high-level use of the DMP in 2012). High-level use was considered as GPs having achieved a median monthly rate (within 1 year) of coding 70% of patient visits in the EHR. Furthermore, to be classified as high-level use, GPs were required to access the disease management module over at least 4 months in their exposure year; this restriction ensured that GP practices were repeatedly using the disease management features of the system. These criteria were interpreted as the GP truly using the DMP. Unexposed patient-years were those associated with patients attending 135 ‘control’ GPs in 2012, which achieved high-level use of the DMP in the following year (i.e. in 2013).

### Statistical analysis

We applied a patient-level logistic regression analysis on whether or not a patient received a lipid-lowering medication within the 12 months following the baseline year. We adjusted for age, sex, diabetes duration, Charlson Comorbidity Index (CCI) scores and psychosocial deprivation (as measured by having either a history of antipsychotic/antianxiety/antidepression medication use [ATC N05A + B + C or ATC N06A] within the 3 years prior to study baseline or being unemployed while below retirement age [<67 years]). Furthermore, heterogeneity of impact of exposure was assessed, considering subgroups based on confounder variables. We report ORs and 95% CIs for the model estimates, using *p* < 0.05 as statistical threshold.

To be confident that the results from the main analysis reflect the patients’ exposure to GP use of the DMP, placebo analyses were conducted, shifting the study period back by 2 years (i.e. to 2010), prior to the national rollout of the EHR/DMP.

## Results

Baseline descriptives are presented in Table 1. Exposed patients were marginally older and had higher CCI scores.

**Table 1:** Baseline (2011) characteristics for patients attending exposed and control GPs

Variable	Attending exposed GPs^a^ (*n* = 5135)	Attending control GPs^b^ (*n* = 3936)	*p* values
GPs (number of clinics)	165	135	
GP list size (number of patients)	2902 ± 1500	2831 ± 1522	0.69
Age, years	61.6 ± 16.7	60.9 ± 16.8	0.03
Diabetes duration, years	6.0 ± 4.5	5.8 ± 4.4	0.06
Male, %	51.0 ± 50.0	51.2 ± 50.0	0.87
CCI score	0.43 ± 1.0	0.39 ± 1.0	0.04
Deprived, %^c^	34.8 ± 47.6)	34.2 ± 47.4	0.55
GP rate of lipid-lowering drug prescriptions received by T2D patients, %	70.6 ± 9.3	70.4 ± 8.7	0.82

Data are reported as mean ± SD, unless stated otherwise

^a^Includes patients with type 2 diabetes who had not received lipid-lowering medications in 2011 and attended GPs that achieved high-level use of the DMP system in 2012

^b^Includes patients with type 2 diabetes who had not received lipid-lowering medications in 2011 and attended GPs that achieved high-level use of the DMP system in 2013

^c^Patients with a history of antipsychotic/antianxiety/antidepression medication (ATC N05A + B + C or ATC N06A) within the 3 years prior to the baseline year, or those unemployed and <67 years of age

T2D, type 2 diabetes

Patients attending GPs that used the DMP at a high level in 2012 had significantly greater odds of receiving lipid-lowering medications when compared with patients who attended GPs that did not use the DMP at a high level before 2013 (OR 1.23 [95% CI 1.09, 1.38; Table 2).

**Table 2:** Results of logistic regression analysis: effect of DMP use on lipid-lowering drug prescribing in 2012

Model	*n*	OR (95% CI)	Adjusted OR (95% CI)^a^
Main model (overall)	9071	1.22 (1.09, 1.37)	1.23 (1.09, 1.38)
Subgroup models			
Age, years			
< 60	3707	1.20 (0.99, 1.45)	1.20 (0.99, 1.45)
60–70	2325	1.40 (1.13, 1.73)	1.40 (1.13, 1.74)
> 70	3039	1.11 (0.90, 1.37)	1.12 (0.91, 1.39)
Diabetes duration, years			
≤ 5	4399	1.33 (1.13, 1.56)	1.33 (1.13, 1.56)
> 5	4672	1.11 (0.94, 1.33)	1.12 (0.94, 1.33)
Sex			
Male	4637	1.30 (1.11, 1.52)	1.32 (1.12, 1.54)
Female	4434	1.13 (0.95, 1.35)	1.14 (0.95, 1.36)
CCI score^b^			
0	7018	1.26 (1.11, 1.44)	1.27 (1.11, 1.45)
1–2	1597	1.21 (0.91, 1.61)	1.21 (0.91, 1.61)
> 2	456	0.71 (0.41, 1.22)	0.74 (0.43, 1.28)
Deprivation			
Not deprived	5912	1.24 (1.07, 1.43)	1.25 (1.08, 1.45)
Deprived	3159	1.18 (0.97, 1.45)	1.20 (0.98, 1.47)

^a^Adjusted ORs are based on logistic regression analyses adjusting for all confounder variables (age, sex, diabetes duration, CCI scores and psychosocial deprivation), excluding any stratification variable

^b^CCI score = 0, no comorbidities; CCI score = 1–2, moderately severe comorbidities; CCI score> 2, highly severe comorbidities

Statistically significant associations of exposure to high-level use of the DMP with lipid-lowering drug prescriptions were identified among specific patient subgroups, indicating where the impact of the system may have been concentrated (Table 2). More specifically, these significant associations were observed in patients in the 60–70 year age range (OR 1.40 [95% CI 1.13, 1.74]), patients with no more than 5 years of diabetes duration (OR 1.33 [95% CI 1.13, 1.56]), male participants (OR 1.32 [95% CI 1.12, 1.54]), patients with a CCI score equal to 0 (i.e. without comorbidities; OR 1.27 [95% CI 1.11, 1.45]) and non-deprived patients (OR 1.25 [95% CI, 1.08, 1.45]).

Upon shifting the analysis period back by 2 years, prior to the national rollout of the DMP, no significant impact of placebo exposure to high-level DMP on lipid-lowering medicine prescribing was identified in the overall or subgroup analyses (electronic supplementary material [ESM] Table 1).

## Discussion

Our study showed that patients with type 2 diabetes with no recent history of lipid-lowering treatment, who were listed with GPs that used a DMP, which provided a population overview with alerts for patient clinical performance, had statistically significantly greater odds of receiving a prescription for lipid-lowering medications when compared with patients who attended GPs who had not started using the DMP at a high level. The increase in lipid-lowering drug prescriptions was especially notable for male patients, patients aged 60–70 years, patients with no more than 5 years of diabetes duration, non-deprived patients and patients with no comorbidities.

A review by Hemens et al [8] concludes that decision-support tools that use alerts or reminders for drug prescribing inconsistently improved process-of-care quality. A study using participants from Kaiser Permanente, in the USA, by Derose et al [9], found a moderate impact on statin prescription to patients with diabetes following an intervention that offered single-page sheets with pertinent patient data, including a recommendation to prescribe the indicated medication, to be faxed to the healthcare provider from a central location on the morning of a scheduled outpatient appointment.

Filippi et al [10] found an effect on antiplatelet drug prescriptions for patients with diabetes following use of an electronic reminder integrated into a computer system that successfully aimed to increase the use of the system among Italian general practitioners. Results were stratified by CVD risk into three groups: (1) one risk factor without CVD; (2) two or more risk factors without CVD; and (3) the presence of at least one type of CVD. The magnitudes of the effects were largest for those without CVD but with one or more risk factor (groups 1 and 2). The results of our stratified analyses are in line with this finding.

The decision-support tool in the context of our study may be defined as weaker than both the US and the Italian tools described above. This is because it did not offer alerts or reminders at the point of service, but only worked through the population overview, which the general practitioner would access on their own initiative. Hence, behavioural changes in our study required additional physician effort. The Italian study showed a much higher impact of the intervention of medication prescription than that observed in our study, which may indicate that further improvements may be gained by using electronic reminders/alerts at the time that physicians and patients meet.

A strength of our study is that it is based on nationwide, high-quality register data, with practically no missing variable bias. A limitation is that our measurement of exposure may be biased by self-selection of GPs with a special interest in diabetes treatment. The risk of selection bias was reduced by omitting the first users of the DMP and using a cohort of GPs that only began using the DMP in 2012 (a year after enrolment became mandatory), by using GPs who fulfilled the exposure criteria during the following year (2013) as a control group and by controlling for a range of confounder variables. Finally, assessment of selection bias by running a placebo test to evaluate differences in outcomes prior to exposure leaves us confident that residual confounding was a minor problem.

## Supplementary information

ESM Table 1 (PDF 122 kb)

## Data Availability

This study is based on micro-data analysed at a server on Statistics Denmark. Data are only available for Danish Research Institutions and cannot be shared outside the server.

## Abbreviations

ATC: Anatomical therapeutic chemical
CCI: Charlson Comorbidity Index
DMP: Disease management program
EHR: Electronic health records
GP: General practice
