# Supplementary material for: Use of electronic patient data overview with alerts in primary care increases prescribing of lipid-lowering medications in patients with type 2 diabetes
Source: Diabetologia. 2021 Oct 28;65(2):286–90. doi: 10.1007/s00125-021-05598-x (PMC8741655; doi:10.1007/s00125-021-05598-x)
Supplement: Supplementary file 1 — (PDF 122 kb) [file 125_2021_5598_MOESM1_ESM.pdf]

Electronic Supplementary Material

ESM Table 1: Results of logistic regressions with a placebo exposure in 2010

	N	Odds Ratio	95% CI	Adjusted Odds Ratio	95% CI
Main model	7,733	1.06	0.93 - 1.20	1.06	0.93 - 1.20
Subgroup models					
Age					
<60	3,020	1.06	0.86 - 1.29	1.06	0.86 - 1.30
60-70	1,978	1.06	0.84 - 1.33	1.06	0.84 - 1.33
>70	2,735	1.08	0.86 - 1.35	1.08	0.86 - 1.35
Diabetes duration					
≤5 years	3,643	1.02	0.85 - 1.21	1.01	0.84 - 1.21
>5 years	4,090	1.11	0.93 - 1.33	1.11	0.93 - 1.32
Gender					
Male	4,018	1.01	0.86 - 1.20	1.02	0.86 - 1.21
Female	3,715	1.11	0.92 - 1.34	1.11	0.92 - 1.35
CCI Score					
CCI:0	5,923	1.06	0.92 - 1.22	1.06	0.92 - 1.23
CCI: 1-2	1,388	1.14	0.85 - 1.52	1.13	0.84 - 1.51
CCI: >2	422	0.83	0.48 - 1.42	0.82	0.48 - 1.42
Deprivation					
Not Deprived	4,958	1.15	0.98 - 1.34	1.15	0.98 - 1.34
Deprived	2,775	0.92	0.75 - 1.13	0.92	0.75 - 1.14

Notes: CCI: Charlson Comorbidity Index. Adjusted Odds Ratios are based on logistic regressions using all confounder variables excluding any stratification variable.
